# Analysis of the impact of a university distance learning course on digitalization in medicine on students and healthcare professionals

**DOI:** 10.1007/s00508-024-02393-7

**Published:** 2024-07-10

**Authors:** Martin Baumgartner, Michaela Wagner-Menghin, Christian Vajda, Gernot Lecaks, Armin Redzic, Georg Dorffner

**Affiliations:** 1 Center for Medical Data Science, Medical University of Vienna, Währinger Straße 25A, 1090 Vienna, Austria; 2 Department of Psychiatry and Psychotherapy, Medical University of Vienna, Vienna, Austria; 3 Department of Medical Psychology, Psychosomatics and Psychotherapy, Medical University of Graz, Graz, Austria; 4 University Clinic for Medical Psychology and Psychotherapy, Medical University of Graz, Graz, Austria

**Keywords:** Curriculum, Digital health, Medical informatics, Education, Surveys and questionnaires

## Abstract

**Purpose:**

The public medical universities in Austria (educating 11,000 students) developed a joint public distance learning series in which clinicians discussed current digital lighthouse projects in their specialty. This study aims to examine the changes in attitude and knowledge of the participants before and after the lecture series to gain insights for future curriculum developments.

**Method:**

The lecture series was announced via various channels at the universities, in health newsletters and in social media. Attitudes toward digitalization in medicine were surveyed before and after the lecture series, together with demographic data. The data were analyzed statistically and descriptively for four groups of interest: female medical students, male medical students, faculty members and members from industry and public agencies.

**Results:**

Out of 351 subjects who attended at least 1 lecture, 117 took part in the survey before and 47 after the lectures. Most participants had a positive attitude towards digitalization (85.3%). They improved their self-assessment of their knowledge from 34.4% to 64.7% (*p* < 0.05). After the lecture series 55.8% of participants considered digital medical applications to be important or very important today and 68.6% in the future.

**Conclusion:**

The study shows that the presentation and discussion of lighthouse projects improves understanding of digitalization in medicine but does not trigger a strong desire for additional further training.

**Supplementary Information:**

The online version of this article (10.1007/s00508-024-02393-7) contains supplementary material, which is available to authorized users.

## Introduction

Digitalization entered the healthcare sector years ago, and the number of digital applications is growing rapidly. This increasing number of future digital health applications can easily be seen in the application process of the US Food and Drug Administration (FDA). In order to submit an application, all supporting clinical studies must be registered in the ClinicalTrials.gov database, an official website of the United States government [1]. A query of this public database for “study start” in the 5‑year period from 1 January 2014 to 1 January 2019, and the 5‑year period from 1 January 2019 to 1 January 2024, showed a steep increase in digital health terms in study records. The number of launched studies where the study records contained the search term “electronic health record” increased from 976 to 1982 from the first period to the second period, the search term “decision support system, clinical” increased from 481 to 890, and most notably, the search term “artificial intelligence” increased from 135 launched studies to 1520. The results of these studies will soon be incorporated into clinical practice.

Empowering all healthcare ecosystem participants with digital skills is the key to unlocking the full potential of digitalization for patient benefit [2, 3]. Future physicians must not only know how to use digital tools [4] but they are also required to bring this knowledge into the healthcare ecosystem [5–7].

Various initiatives of the European Union [8], individual member states of the European Union [9–11] and representative bodies [12, 13] are addressing the development of digital skills of healthcare professionals. Medical universities are updating their curricula [14–20]. Austria’s public medical universities (Medical University of Vienna, Medical University of Graz, Medical University of Innsbruck) and the Medical Faculty of Johannes Kepler University (JKU) with a total of 11,000 students have joined forces and set up the project “Digital skills, knowledge and communication for medical students” to develop a new curriculum module on digitalization in medicine [21]. As part of this initiative, Baumgartner et al. [22] found that medical students’ current knowledge of digitalization in medicine was limited to popular topics from the press, and that they were, in general, sceptical about digitalization in medicine. Furthermore, Körner and Seufert [23] have shown that lecturers are still unsure and unprepared in this new area. With the aim of arousing curiosity and interest in digitalization and creating a pull for digitalization in medical training, a series of lectures entitled “Health 4.0—Digital transformation in healthcare” was developed as part of the overall project and made available to students, faculty and healthcare professionals as a distance learning course.

This paper examines the different knowledge and attitudes of female medical students, male medical students, faculty, and members from industry and public agencies before and after the lecture series to provide inputs for lecturer training and curriculum developer.

## Methods

### Study ethics approval and consent to participate

The ethics committee of the Medical University Vienna deemed this study exempt from requiring a formal decision. The data security board of the Medical University Vienna gave its approval to this study. Participation was voluntary. Participants gave their consent on the first page of the questionnaire.

### Study inclusion criteria and recruitment

All registered participants in the lecture series were eligible to attend. The lecture series was communicated to all four participating medical universities via student newsletters. Various communication channels were used to reach healthcare professionals, including the faculty members’ newsletter, invitation mailings and posts on various social platforms.

### Lecture series concept

The public Austrian medical universities and the Faculty of Medicine at the JKU Linz jointly organized the public learning series “Health 4.0—Digital transformation in healthcare”. The lecture series was delivered as an online distance learning program to ensure broad accessibility. As a low threshold learning opportunity, it is aimed at students, faculty and healthcare professionals to arouse curiosity and interest in digitalization and make a significant contribution to understanding the effects of digitalization in medicine.

All project team members identified outstanding digital projects at the different universities to present the latest applications and processes related to digitalization in medicine and to share real-world experiences from various medical specialties. The selected lighthouse projects were: “Robotic in ear, nose and throat (ENT) surgery”, “Digitalisation in pathology”, “Digital public health”, “Connected devices, data and security”, “Digital eye clinic”, “Real-world data in clinical research”, “Digital patient journey, Austrian electronic health records (ELGA), eHealth and digital therapeutics”, “Implantable heart monitor in heart attack aftercare”, “The digital patient consultation”, “Digital pills”, “Digital solutions for mental health”, “Mixed reality in operating room simulation” and “Genomic medicine and molecular precision medicine”. Each lecture consisted of a 1h presentation followed by a 30min discussion. Lecturers were renowned clinicians and industry specialists.

### Study design

The survey was based on previously published surveys [24, 25] and adjusted to the specific requirements of the study by an expert panel consisting of a physician, a computer scientist, two educational experts, a medical student and a technology expert. The survey covers the areas of demographics, knowledge self-confidence in digital applications, attitudes towards the future impact of digital medicine and educational requirements for the curriculum. In total 17 questions were defined, 8 questions to collect demographic data and 9 questions with subitems, each subitem based on 5‑point Likert scales for the various areas of interest (Appendix 1). The Likert scales were defined with 1 = “not at all”, 2 = “a little”, 3 = “moderately”, 4 = “fairly”, 5 = “very much” and analogously applies “not at all important”, “not very important”, “moderately important”, “important”, “very important”. A total of 16 participants were recruited for the pretest and provided comments on clarity, ease of use, and general issues. Recruitment for the pretest was done through personal contacts. As part of the internal review process, feedback was discussed, agreed and incorporated into the questionnaire by two researchers. The survey was deployed as a web-based survey using the SociSurvey platform (SW Version 3.2.11, SoSci Survey GmbH, Munich, Germany). The same survey was conducted at the beginning of the lecture series (prelecture survey) and at the end of the lecture series (postlecture survey).

The study and the survey were written in German to consider the fact that the medical curricula at Austrian universities are taught in German. The questions were translated for this manuscript.

### Data collection

To ensure participant anonymity, invitations to both the prelecture and postlecture surveys were sent to all 440 registered participants. Participants were redirected to the first page of the survey on the SociSurvey website via an embedded email link. Upon providing informed consent on the first page, participants were able to access and begin the survey.

### Data analysis

Participants selected one of seven professional groups. For the analysis, the professional groups were combined into four groups: female medical students, male medical students, faculty, industry professionals and health agency representatives.

During data cleansing, data records containing only demographic data were excluded. The data were analyzed using statistical software IBM SPSS Statistics for Windows, Version 25.0. Armonk, NY: IBM Corp., whereby a *p*-value less than 0.05 was considered significant. After analyzing skewness and kurtosis the assumption of normal distribution was rejected and nonparametric tests were used. A Mann-Whitney U test was used to check whether the central tendencies of the pretest and the posttest differ. The tendencies for the questions “Please rate the current importance of the following applications within your (intended) specialty.” and “Please estimate which digital applications will be particularly important in the next 5 years within your (intended) specialty.” were analyzed with a paired Wilcoxon test for the prelecture data and for the postlecture data. If a question consisted of subitems, the *p*-value was calculated only for the item itself. Cronbach’s alpha was used to test internal consistency. A Cronbach’s alpha of 0.824 indicates good internal consistency.

For the descriptive analysis, the results of the two highest or most positive categories on the 5‑point Likert scale were added together, i.e. “fairly”/“very much” or “important”/“very important” were added together, and a threshold value of 80% was agreed and set to improve the presentation of the results.

## Results

### Demographics

A total of 440 participants registered for the distance learning course series and 351 registered participants took part in the lecture series at least once.

The invitation to participate was sent out for the prelecture survey and the postlecture survey to all registered participants via an invitation email, 117 and 47 responses were received (response rate 33.3% and 13.4%, respectively). After data cleansing 101 and 41 responses were used for the analyses. Table 1 shows the sociodemographic characteristics of the survey participants. The majority were male medical students (42.6% and 31.7%). The ratio between female and male medical students is constant for both surveys (prelecture survey: 45.5%/53.5%, postlecture survey: 46.3%/53.7%), which differs from the total Austrian medical student population (53.8%/46.2%).

**Table 1:** Sociodemographic characteristics of the survey participants

	Prelecture survey		Postlecture survey	
	*n*	%	*n*	%
*Status*				
Female medical students	29	28.7	9	22.0
Male medical students	43	42.6	13	31.7
Faculty members	18	17.8	12	29.3
Industry or public agency	11	10.9	7	17.1
Total	101	–	41	–
*Gender*				
Female	46	45.5	19	46.3
Male	54	53.5	22	53.7
Undeclared	1	1.0	0	0.0
Total	101	–	41	–
*Age group (years)*				
<21	8	7.9	2	4.9
21–25	53	52.5	16	39.0
26–30	17	16.8	5	12.2
31–40	8	7.9	9	22.0
41–50	5	5.0	4	9.8
>50	10	9.9	4	9.8
Undeclared	0	0.0	1	2.4
Total	101	–	41	–

### General interest in digitalization in medicine

In this study three statements were used to understand the interests of the participants towards digitalization, “I enjoy working with digital applications”, “Digitalization makes my work more efficient”, “I am interested in using digital products in clinical routine”. In the prelecture survey in each of the 4 groups more than 80.0% agreed or strongly agreed. In the postlecture survey, the results moved towards 90.0%, except for faculty members where the agreement for the statement “Digitalization makes my work more efficient” dropped to 60.0%.

### Knowledge self-assessment

For self-assessment the participants answered the question “How satisfactorily can you explain the following terms to an interested layperson?” (The full list of items is shown in Appendix 1).

**Fig. 1:**
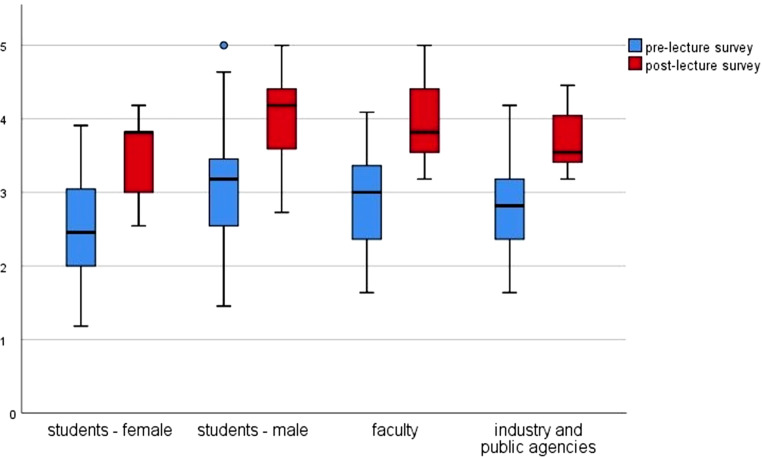
Knowledge self-assessment “How satisfactorily can you explain the following terms to an interested layperson” Boxplot showing the improvements in knowledge of the four groups analyzed before (prelecture survey) and after (postlecture survey) the lecture series, measured using a 5-point Likert scale (*Y‑axis*)

In the prelecture survey 34.4% of all participants answered the subitems with “fairly” or “very much”. This value increased to 64.7% in the postlecture survey (*p* < 0.05). Female medical students feel much less confident than the other groups, with their results rising from 21.9% to 47.5%. Male medical students, on the other hand, feel much more confident, with their results rising from 42.1% to 70.2%, which is in line with current scientific findings [26, 27]. The results for faculty member rose from 35.7% to 70.2% and for the participants from industry and public agencies from 33.3% to 67.5%, respectively. Details see Fig. 1.

### Attitude towards the future development of digitalization in medicine

Two questions were analyzed in this section: “Please rate the current importance of the following applications within your (intended) specialty.” and “Please estimate which digital applications will be particularly important in the next 5 years within your (intended) specialty.” (The full list of items is shown in Appendix 1).

In the prelecture survey, the Wilcoxon test showed a significant difference (*p* < 0.05) between current importance and importance in 5 years. The postlecture survey showed a much higher rating of current importance of digitalization in medicine and a similar importance in 5 years resulting in a nonsignificant (*p* = 0.15) result.

In the prelecture survey the four groups in total rated “the current importance” of 8 applications above the threshold, where “telemonitoring”, “medical databases” and “big data” were rated by 2 groups, 13 applications were rated as “will be particularly important within 5 years”, where “digital imaging”, “telemedicine”, “medical database” and “big data” were rated by 2 or more groups.

In the postlecture survey, the 4 groups in total rated “the current importance” of 11 applications above the threshold, where “digital diagnostics”, “digital imaging”, “medical databases” and “big data” were rated by 2 or more groups. Of the applications 18 were rated as “will be particularly important in 5 years”. “digital diagnostics”, “digital imaging”, “telemedicine”, “medical database” and “big data” were rated by 2 or more groups.

### Attitude towards the future importance of digitalization in medicine for various specialties

Question 3 prompted participants to estimate “Which medical specialty will particularly benefit from digitalization in the future?” (All options are shown in Appendix 1).

In the prelecture survey “medical genetics” were rated by all four examined groups above the threshold. “Radiation therapy-radiation oncology” was rated by three groups. There was no specialty rated by two groups. Female medical students, male medical students and faculty members identified only two out of 19 specialties as most likely to experience significant benefits from digitalization in medicine.

In the postlecture survey nine specialties were identified by a minimum of two groups. “Surgical special subjects” were identified by all four groups. “Medical genetics”, “Pharmacology and toxicology” and “public health” were identified by three groups. “Ophthalmology and optometry”, “neurology”, “Radiation therapy-radiation oncology”, “radiology” and “nuclear medicine” were identified by two groups. The female medical students identified seven specialties, while the male medical students identified five specialties. Faculty members continued to name two specialties, which represents a relatively low expectation of the possibilities of digitalization in medicine. Members of industry and public agencies are the most positive group, naming nine specialties as most likely to be positively impacted by digitalization.

### Attitude towards education in undergraduate trainings

The participants got four questions to rate: “Should informatic basics be part of the compulsory curriculum?”, “Should knowledge about specific digital applications be part of the compulsory curriculum?”, “Should lectures about digitalization in medicine only be elective?” and “There is no need for additional training, digital knowledge from the high school is sufficient”.

In the prelecture survey 75.0% of all participants consented that informatics basics should be part of the compulsory curriculum and 80.7% consented knowledge about concrete digital application should be part of the compulsory curriculum. The group with the highest support for digitalization in the compulsory curriculum were members of the industry and public agencies (100.0%, 100.0%, respectively). The group with the least support were male medical students (68.4%, 68.4%). The results for female medical students (88.5%, 76.9%) and faculty members (87.5%, 75.0%) were close to each other.

In the postlecture survey, 73.0% of all participants consented that informatics basics should be part of the compulsory curriculum and 67.6% consented knowledge about concrete digital application should be part of the compulsory curriculum. Male medical students were again the group with the least support (53.8%, 61.5%) and 23.1% of male medical students consented that elective lectures or high school training is sufficient. Female medical students were more interested in compulsory basics than application trainings (100.0%, 75.0%), whereas members of the industry and public agencies rated applications higher than basics (83.3%, 100.0%). Faculty members (60.0%, 60.0%) were similar to male medical students, but 40.0% of faculty members selected electives are sufficient training for future physicians.

## Discussion

The number of digital applications within the medical field will increase dramatically over the next few years. Moreover, patients will use more and more lifestyle products that collect patient data 24 h a day. This will change the way physicians do their work.

Many universities have taken initiatives to include lectures on digitalization in medicine in their curriculum. In a survey in Germany, Aulenkamp et al. [15] identified 16 medical faculties who offer lectures in digitalization in medicine. Tudor et al. [17] conducted a systematic review and identified 34 existing courses, including two courses from Germany. The reported digital health courses were mostly elective, were integrated into the existing curriculum, covered medical informatics and provided a general overview of current digital trends in healthcare, such as digital health applications and robotics.

In all these courses, digitalization in medicine was treated as a sum of additional tools. To realise the full potential of digitalization in medicine, it is essential to introduce data-driven thinking and provide a connected learning experience with different disciplines. For instance, patient monitoring is evolving from intermittent, in-person visits to continuous data collection, enabling physicians to access patient information in real-time, provide more in-depth information about treatment success and adherence, and adjust treatments more easily and quickly. With the rapid advances in digital technology, its importance in medicine is becoming comparable to traditional disciplines like biology, chemistry and physics. Digitalization in medicine will change standard operating procedures (SOPs) and medical guidelines.

As part of the project “Digital skills knowledge and communication for medical students” Baumgartner et al. [22] found scepticism among the students. Körner and Seufert [23] also found that faculties are not well prepared and reluctance to embrace digitalization in medicine. To accelerate the acceptance of digitalization among students and faculties, a positive approach to digitalization in medicine is required. This study took the approach of presenting and discussing digital lighthouse projects in medicine to arouse curiosity and increase interest in this new field. After the lecture series all examined groups, with the exception of faculty members, acknowledged the broad opportunities of digitalization in medicine in various specialties; however, the differences between the prelecture survey and postlecture survey for the need of training did not reflect the broader knowledge. Female medical students in both surveys voted mainly for lectures within the compulsory curriculum. Male medical students and faculties also voted for electives or even stated that the high school training is sufficient. This leads to the conclusion that although participants are interested in digitalization in medicine, it is still seen as an additional tool rather than a new way of treating patients and organizing healthcare.

The number of participants exceeded the expectations of the organizing universities. Based on these results, the universities developed various electives in which physicians, computer scientists and medical engineers work together. In addition, existing courses were enriched with topics related to digitalization in medicine. In order to remedy the lack of experienced lecturers with digitalization knowledge in the medical curriculum, the Medical University of Vienna relies on lecturers from the master’s degree program in medical informatics and physicians with a technical background. The Medical University of Graz draws on lecturers from the Institute of Medical Informatics, Statistics and Documentation as well as interested physicians. At the Medical University of Innsbruck, a professorship for “digitalization” is in preparation. The Faculty of Medicine at the JKU Linz draws on lecturers from the master’s degree program in medical engineering [21].

## Limitations

Only participants in the lecture series were included in this study. This approach is likely to introduce selection bias, as participants who opted for the series might already hold a more positive attitude towards digitalization in medicine compared to a representative sample.

To ensure participant anonymity, invitations to both the prelecture and postlecture survey were sent to all registered participants. Consequently, it is possible that not only participants who completed the prelecture survey also completed the postlecture survey. No information was collected on how many, and which lectures were attended by the participants in the postlecture survey. This can lead to a further selection bias.

## Conclusion

This study offers a better understanding of the curriculum development needs for digitalization in medicine for medical universities. Particularly important are the differing digital health needs of female and male medical students. The study also reveals a reluctance of faculty members to embrace digitalization in medicine. Beyond developing teaching content, medical universities must also consider realigning curriculum requirements for future physicians [28, 29]. This realignment should aim to create a learning environment that fosters a positive attitude towards the potential of digitalization in medicine. Further research is needed to investigate the different outcomes of female and male medical students and to identify the reasons for faculty reluctance to embrace digitalization.

Based on the high number of participants in the lecture series and the results of the study, the described lecture series fulfilled its objective as a low-threshold educational offering, fostering knowledge about digitalization in medicine among various members of the healthcare ecosystem.

## Supplementary Information

Appendix 1 Questionnaire

## References

[1] ClinicalTrial.gov. https://www.clinicaltrials.gov/. Accessed 29 Feb 2024.

[2] Barakat A, Woolrych RD, Sixsmith A, Kearns WD, Kort HS. ehealth technology competencies for health professionals working in home care to support older adults to age in place: outcomes of a two-day collaborative workshop. Med 20. 2013;2(2):e10. 25075233 10.2196/med20.2711 PMC4084768

[3] Wartman SA, Combs CD. Medical education must move from the information age to the age of artificial intelligence. Acad Med. 2018;93(8):1107–9. 29095704 10.1097/ACM.0000000000002044

[4] Hsiang EY, Ganeshan S, Patel S, Yurkovic A, Parekh A. Training physicians in the digital health era: how to leverage the residency elective. JMIR Med Educ. 2023;9:e46752. 37450323 10.2196/46752 PMC10383775

[5] Greenberg AJ, Haney D, Blake KD, Moser RP, Hesse BW. Differences in access to and use of electronic personal health information between rural and urban residents in the United States. J Rural Health. 2018; 10.1111/jrh.12228. 28075508 10.1111/jrh.12228 PMC5505819

[6] Culler SD, Atherly A, Walczak S, Davis A, Hawley JN, Rask KJ, et al. Urban-rural differences in the availability of hospital information technology applications: a survey of Georgia hospitals. J Rural Health. 2006;22(3):242–7. 16824169 10.1111/j.1748-0361.2006.00039.x

[7] Matusiewicz D, Aulenkamp J, Werner AJ. Effekte der digitalen Transformation des Krankenhauses auf den Wandel des Berufsbildes Arzt. In: Klauber J, Geraedts M, Friedrich J, Wasem J, editors. Krankenhaus-Report 2019. Berlin, Heidelberg: Springer; 2019. pp. 101–14. 10.1007/978-3-662-58225-1_8.

[8] Consensus framework on the digital transformation of healthcare. https://www.cpme.eu/api/documents/adopted/2021/7/Info.2021-096.Consensus.Framework.pdf. Accessed 29 Feb 2024.

[9] Digital Austria Act. Bundeskanzleramt, Bundesministerium Kunst, Kultur, öffentlicher Dienst und Sport, Bundesministerium Finanzen. 2023. https://www.digitalaustria.gv.at/dam/jcr:65838693-199c-43a0-92da-ffa4a8791f50/MRV%20Digital%20Austria%20Act%20DAA-61_10_Hauptdok.pdf. Accessed 24 July 2023.

[10] Preparing the healthcare workforce to deliver the digital future. 2019. https://topol.hee.nhs.uk/wp-content/uploads/HEE-Topol-Review-2019.pdf. Accessed 17 Oct 2023.

[11] Federal Ministry of Health. Digital together—Germany’s digitalisation strategy for health and care. https://www.bundesgesundheitsministerium.de/fileadmin/Dateien/3_Downloads/D/Digitalisierungsstrategie/Germany_s_Digitalisation_Strategy_for_Health_and_Care.pdf. Accessed 29 Feb 2024.

[12] Michel A, Baumgartner P, Brei C, Bullingerr-Hoffmann A, Gerdes A, Hesse F, et al. Diskussionspapier: Framework zur Entwicklung von Curricula im Zeitalter der digitalen Transformation [Internet]. hochschulforum digitalisierung. https://hochschulforumdigitalisierung.de/sites/default/files/dateien/Diskussionspapier1_Framework_Curriculumentwicklung.pdf. Accessed 24 July 2023.

[13] Brandt J, Frey N. Digitale Transformation in der medizinischen Ausbildung. https://hochschulforumdigitalisierung.de/wp-content/uploads/2023/11/HFD_AP_74_Medizin.pdf. Accessed 29 Feb 2024.

[14] Rieder A. Ausbildung zukünftiger Ärztinnen und Ärzte im Zusammenhang mit der digitalen Transformation und COVID-19 als Herausforderer im Medizinstudium. Zgp – Z Gesundheitspolitik. 2020;02:61–83.

[15] Aulenkamp J, Mikuteit M, Löffler T, Schmidt J. Overview of digital health teaching courses in medical education in Germany in 2020. GMS J Med Educ. 2021;38(4):Doc80. [cited 2023 Jul 24]; https://www.egms.de/en/journals/zma/2021-38/zma001476.shtml. 34056069 10.3205/zma001476 PMC8136344

[16] Werner R, Henningsen M, Schmitz R, Guse AH, Augustin M, Gauer T. Digital Health meets Hamburg integrated medical degree program iMED: concept and introduction of the new interdisciplinary 2nd track Digital Health. GMS J Med Educ. 2020;37(6):Doc61. [cited 2023 Jul 24]; Available from: https://www.egms.de/en/journals/zma/2020-37/zma001354.shtml. 33225053 10.3205/zma001354 PMC7672380

[17] Car TL, Kyaw BM, Nannan Panday RS, Van Der Kleij R, Chavannes N, Majeed A, et al. Digital health training programs for medical students: scoping review. JMIR Med Educ. 2021;7(3):e28275. 34287206 10.2196/28275 PMC8339984

[18] Hautz SC, Hoffmann M, Exadaktylos AK, Hautz WE, Sauter TC. Digital competencies in medical education in Switzerland: an overview of the current situation. GMS J Med Educ. 2020;37(6):Doc62. [cited 2023 Jul 24]; https://www.egms.de/en/journals/zma/2020-37/zma001355.shtml. 33225054 10.3205/zma001355 PMC7672378

[19] Aungst TD, Patel R. Integrating digital health into the curriculum—considerations on the current landscape and future developments. J Med Educ Curric Dev. 2020;7:238212051990127. 10.1177/2382120519901275 PMC6971961 32010795

[20] Universität Zürich. White Paper Curriculumsrevision ZH Med4 für Themenblöcke der Studienjahre 3–4 [Internet]. Universität Zürich,, Medizinische Fakultät. 2019. https://www.med.uzh.ch/dam/jcr:476b3cd6-a905-4d68-b766-f83fb303edd8/2022_Stand%2020220729_WhitePaper_ZHMed4_Themenbloecke_SJ3-4_yb.pdf..

[21] Baumgartner M, Fazekas C, Simonic KM, Vajda C, Michlmayr-Brand K, Lecaks G, et al. The “digital skills, knowledge and communication for medical students” project. https://zfhe.at/index.php/zfhe/article/view/1763. Accessed 29 Feb 2024.

[22] Baumgartner M, Sauer C, Blagec K, Dorffner G. Digital health understanding and preparedness of medical students: a cross-sectional study. Med Educ Online. 2022;27(1):2114851. 36036219 10.1080/10872981.2022.2114851 PMC9423824

[23] Körner J, Seufert T. Wie digital kompetent sind Lehrende? Erfassung und Förderung digitaler Kompetenzen von Hochschullehrenden der Humanmedizin. In: Gemeinsame Jahrestagung der Gesellschaft für Medizinische Ausbildung (GMA) und des Arbeitskreises zur Weiterentwicklung der Lehre in der Zahnmedizin (AKWLZ); Halle. Düsseldorf: German Medical Science GMS Publishing House; 2022.

[24] Vossen K, Rethans JJ, Van Kuijk SMJ, Van Der Vleuten CP, Kubben PL. Understanding medical students’ attitudes toward learning ehealth: questionnaire study. JMIR Med Educ. 2020;6(2):e17030. 33001034 10.2196/17030 PMC7563623

[25] Pinto Dos Santos D, Giese D, Brodehl S, Chon SH, Staab W, Kleinert R, et al. Medical students’ attitude towards artificial intelligence: a multicentre survey. Eur Radiol. 2019;29(4):1640–6. 29980928 10.1007/s00330-018-5601-1

[26] Haug SR, Linde BR, Christensen HQ, Vilhjalmsson VH, Bårdsen A. An investigation into security, self-confidence and gender differences related to undergraduate education in Endodontics. Int Endodontic J. 2021;54(5):802–11. 10.1111/iej.13455 33253460

[27] Casale S. Gender differences in self-esteem and self-confidence. In: Carducci BJ, Nave CS, Nave CS, editors. The Wiley encyclopedia of personality and individual differences. 1st ed. Wiley; 2020. pp. 185–9. 10.1002/9781118970843.ch208.

[28] Meskó B, Drobni Z, Bényei É, Gergely B, Győrffy Z. Digital health is a cultural transformation of traditional healthcare. mHealth. 2017;3:38–38. 29184890 10.21037/mhealth.2017.08.07 PMC5682364

[29] Mesko B, Győrffy Z. The rise of the empowered physician in the digital health era: viewpoint. J Med Internet Res. 2019;21(3):e12490. 30912758 10.2196/12490 PMC6454334

