# Supplementary material for: Analysis of the impact of a university distance learning course on digitalization in medicine on students and healthcare professionals
Source: Wien Klin Wochenschr. 2024 Jul 10;137(13-14):412–8. doi: 10.1007/s00508-024-02393-7 (PMC12241288; doi:10.1007/s00508-024-02393-7)
Supplement: Supplementary file 1 — Appendix 1 Questionnaire [file 508_2024_2393_MOESM1_ESM.pdf]

health → base 01.03.2024, 11:49

Seite 01

Sehr geehrte Teilnehmende der Vortragsreihe *„Health 4.0 – Digitale Transformation im Gesundheitswesen“,*

im BMBWF Projekt: *"Digital Skills, Knowledge & Communication für Studierende der Medizin"* wird daran gearbeitet, Inhalte der Medizincurricula der österreichischen öffentlich-rechtlichen medizinischen Universitäten und Fakultäten an die wachsende Bedeutung der Digitalisierung in der Medizin anzupassen und digitale Lehrmittel zu entwickeln.

Um mehr über Ihre Einstellung zu den Möglichkeiten, Chancen und Risiken der digitalen Medizin zu erfahren, laden wir Sie zu Beginn und am Ende der Vortragsreihe zur Teilnahme an unserer Umfrage ein. Die Teilnahme an der Umfrage erfolgt freiwillig. Die Ablehnung der Teilnahme oder ein vorzeitiges Beenden der Umfrage hat keine nachteiligen Folgen für Sie.

Die Erhebung der Daten erfolgt anonym, ausschließlich für wissenschaftliche Zwecke. Das Studienprotokoll wurde durch die inneruniversitäre Datenschutzkommission der MedUniWien begutachtet und befürwortet.

Durch das Bearbeiten der Umfrage willigen Sie zur Teilnahme an der Studie ein.

Vielen Dank für Ihre Teilnahme!

Für das Projektteam
Kontakt: dhealth@meduniwien.ac.at

Seite 02

Fragebogen „Health 4.0 – Digitale Transformation im Gesundheitswesen"

Begleitend zur Vortragsreihe *„Health 4.0 – Digitale Transformation im Gesundheitswesen“* führen wir eine Untersuchung zu Einstellungen und Erwartungen zur Digitalisierung in der Medizin durch. Dazu bitten wir Sie, sowohl am Beginn als auch am Ende der Veranstaltungsserie, einen Fragebogen zu bearbeiten.

Im folgenden Fragebogen bearbeiten Sie Fragen zu den Themen soziodemographischer Hintergrund und digitaler Anwendungen in der Medizin.

Seite 03

SD01

1. Haben Sie die Vortragsreihe schon einmal besucht?

- ○ Ja
- ○ Nein

SD02

2. Wie alt sind Sie?

- ○ Bis 20
- ○ 21-25
- ○ 26-30
- ○ 31-40
- ○ 41-50
- ○ Ab 51

- ○ Keine Angabe

SD03

3. Welchem Geschlecht fühlen Sie sich zugehörig?

- ○ Weiblich
- ○ Männlich
- ○ Inter
- ○ Divers
- ○ Offen

- ○ Keine Angabe

Seite 04

SD04

4. Welcher Berufsgruppe gehören Sie an?

- Studierende Medizin
- Studierende anderer Fächer (Uni, FH)
- universitäres ärztliches Personal, medizinische Fachkraft
- Nicht-universitäres ärztliches Personal, medizinische Fachkraft
- Wissenschaftliches Personal (Uni, FH)
- Nicht-wissenschaftliches Personal (Uni, FH)
- Personal in der Privatwirtschaft oder in der öffentlichen Verwaltung

5 aktive(r) Filter

Filter SD04/F1
Wenn eine der folgenden Antwortoption(en) ausgewählt wurde: **1**
Dann Frage/Text **SD05** später im Fragebogen anzeigen (sonst ausblenden)

Filter SD04/F2
Wenn eine der folgenden Antwortoption(en) ausgewählt wurde: **1, 2, 5, 6**
Dann Frage/Text **SD06** später im Fragebogen anzeigen (sonst ausblenden)

Filter SD04/F3
Wenn eine der folgenden Antwortoption(en) ausgewählt wurde: **1, 3, 4, 5, 7**
Dann Frage/Text **DG04** später im Fragebogen anzeigen (sonst ausblenden)

Filter SD04/F4
Wenn eine der folgenden Antwortoption(en) ausgewählt wurde: **1, 3, 4, 5, 7**
Dann Frage/Text **DG05** später im Fragebogen anzeigen (sonst ausblenden)

Filter SD04/F5
Wenn eine der folgenden Antwortoption(en) ausgewählt wurde: **1, 3**
Dann Frage/Text **DG09** später im Fragebogen anzeigen (sonst ausblenden)

Seite 05

SD05

5. In welchem Semester befinden Sie sich?

- Semester 1-8
- Ab 9 Semester

Seite 06

SD06

6. Wo sind Sie tätig/inskribiert?

- Medizinische Universität Wien
- Medizinische Universität Innsbruck
- Medizinische Universität Graz
- Medizinische Fakultät der JKU Linz
- andere Ausbildungseinrichtung (Uni, FH)
- Keine Angaben

Seite 07

SD07

7. In welcher medizinischen Fachrichtung oder mit welchem medizinischen Schwerpunkt sind Sie derzeit tätig oder würden später bevorzugt tätig werden wollen?

[Bitte auswählen]

Seite 08

DG01

8. Wie zufriedenstellend können Sie einem interessierten Laien (z.B. Ihrem 16-jährigen Neffen, Ihrer Schwester, ...) die folgenden Begriffe in wenigen Sätzen erklären?

	Gar nicht	Wenig	Mittelmäßig	Ziemlich	Sehr	Keine Angabe
Künstliche Intelligenz	○	○	○	○	○	○
Maschinelles Lernen	○	○	○	○	○	○
Bildformate	○	○	○	○	○	○
Biosignale	○	○	○	○	○	○
Wearables	○	○	○	○	○	○
Internet of Things	○	○	○	○	○	○
Cybersecurity	○	○	○	○	○	○
Krankenhausinformationssystem	○	○	○	○	○	○
Chirurgisches Unterstützungssystem	○	○	○	○	○	○
Intelligente Prothese	○	○	○	○	○	○
Digitale Therapeutika	○	○	○	○	○	○

Seite 09

DG02

9. Bitte schätzen Sie ein, welche digitalen Anwendungen in den nächsten 5 Jahren besonders wichtig in der Medizin sein werden!

	Gar nicht wichtig	Wenig wichtig	Mittelmäßig wichtig	Ziemlich wichtig	Sehr wichtig	Keine Angabe
Robotik in der Chirurgie	○	○	○	○	○	○
Robotik in der Pflege	○	○	○	○	○	○
Digitale Diagnostik	○	○	○	○	○	○
Digitale Bildgebung	○	○	○	○	○	○
Telemedizin	○	○	○	○	○	○
Telemonitoring	○	○	○	○	○	○
Medizinische Datenbanken	○	○	○	○	○	○
Big Data	○	○	○	○	○	○
Patient:innen-Kommunikation über digitale Kanäle	○	○	○	○	○	○
Digitale Therapeutika	○	○	○	○	○	○
Augmented Reality/Virtual Reality	○	○	○	○	○	○

Seite 10

DG03

10. Bitte schätzen Sie ein, welche medizinischen Fachrichtungen in der Zukunft besonders von der Digitalisierung in der Medizin profitieren werden

	Gar nicht	Wenig	Mittelmäßig	Ziemlich	Sehr	Keine Angabe
Allgemeinmedizin	○	○	○	○	○	○
Anästhesiologie und Intensivmedizin	○	○	○	○	○	○
Augenheilkunde und Optometrie	○	○	○	○	○	○
Chirurgische Sonderfächer	○	○	○	○	○	○
Frauenheilkunde und Geburtshilfe	○	○	○	○	○	○
Hals-, Nasen- und Ohrenheilkunde	○	○	○	○	○	○
Haut- und Geschlechtskrankheiten	○	○	○	○	○	○
Internistische Sonderfächer	○	○	○	○	○	○
Kinder- und Jugendheilkunde	○	○	○	○	○	○
Klinisch-Pathologische Sonderfächer	○	○	○	○	○	○
Medizinische Genetik	○	○	○	○	○	○
Neurologie	○	○	○	○	○	○
Orthopädie und Traumatologie	○	○	○	○	○	○
Pharmakologie und Toxikologie	○	○	○	○	○	○
Psychiatrie, Kinder- und Jugendpsychiatrie und Psychotherapeutische Medizin	○	○	○	○	○	○
Public Health	○	○	○	○	○	○
Strahlentherapie-Radioonkologie, Radiologie, Nuklearmedizin	○	○	○	○	○	○
Urologie	○	○	○	○	○	○
Zahnmedizin	○	○	○	○	○	○

Seite 11

DG04

11. Bitte schätzen Sie die derzeitige Wichtigkeit der folgenden Anwendungen für Digitalisierung in der Medizin innerhalb Ihrer (angestrebten) Fachrichtung oder Ihrem medizinischen Schwerpunkt ein.

	Gar nicht wichtig	Wenig wichtig	Mittelmäßig wichtig	Ziemlich wichtig	Sehr wichtig	Keine Angabe
Robotik in der Chirurgie	○	○	○	○	○	○
Robotik in der Pflege	○	○	○	○	○	○
Digitale Diagnostik	○	○	○	○	○	○
Digitale Bildgebung	○	○	○	○	○	○
Telemedizin	○	○	○	○	○	○
Telemonitoring	○	○	○	○	○	○
Medizinische Datenbanken	○	○	○	○	○	○
Big Data	○	○	○	○	○	○
Patient:innen-Kommunikation über digitale Kanäle	○	○	○	○	○	○
Digitale Therapeutika	○	○	○	○	○	○
Augmented Reality/Virtual Reality	○	○	○	○	○	○

Seite 12

DG05

12. Bitte schätzen Sie ein, welche Wichtigkeit den folgenden Anwendungen für Digitalisierung in der Medizin innerhalb Ihrer (angestrebten) Fachrichtung oder Ihrem medizinischen Schwerpunkt innerhalb der nächsten 5 Jahre zukommen werden.

	Gar nicht wichtig	Wenig wichtig	Mittelmäßig wichtig	Ziemlich wichtig	Sehr wichtig	Keine Angabe
Robotik in der Chirurgie	○	○	○	○	○	○
Robotik in der Pflege	○	○	○	○	○	○
Digitale Diagnostik	○	○	○	○	○	○
Digitale Bildgebung	○	○	○	○	○	○
Telemedizin	○	○	○	○	○	○
Telemonitoring	○	○	○	○	○	○
Medizinische Datenbanken	○	○	○	○	○	○
Big Data	○	○	○	○	○	○
Patient:innen-Kommunikation über digitale Kanäle	○	○	○	○	○	○
Digitale Therapeutika	○	○	○	○	○	○
Augmented Reality/Virtual Reality	○	○	○	○	○	○

Seite 13

DG06

13. Beurteilen Sie bei den folgenden Aussagen, wie stark Sie diesen zustimmen.

	stimme gar nicht zu	stimme wenig zu	stimme mittelmäßig zu	stimme überwiegend zu	stimme völlig zu	Keine Antwort
Ich möchte immer die aktuellen Entwicklungen in der Digitalisierung nutzen	○	○	○	○	○	○
Digitalisierung macht mich in meiner Tätigkeit effizienter	○	○	○	○	○	○
Ich genieße die Herausforderungen beim Erlernen neuer digitaler Anwendungen	○	○	○	○	○	○
Es macht keinen Sinn neue digitale Anwendungen einzusetzen, wenn die bestehenden Abläufe funktionieren	○	○	○	○	○	○
Die Herausforderungen bei der Implementierung neuer digitaler Anwendungen sind es nicht wert, bestehende Abläufe zu ersetzen	○	○	○	○	○	○
Digitale Anwendungen fallen immer zum schlechtest möglichen Zeitpunkt aus	○	○	○	○	○	○
Bei aller Digitalisierung, die menschliche Interaktion ist sehr wichtig bei einer medizinischen Tätigkeit	○	○	○	○	○	○
Digitalisierung ist immer bedrohlich, da es bestehendes Wissen und Erfahrung von Personen obsolet macht	○	○	○	○	○	○
Ich habe Spaß an der Arbeit mit digitalen Anwendungen	○	○	○	○	○	○
Die Möglichkeit, neueste digitale Anwendungen zu nutzen hat meine Entscheidung für meine derzeitige Fachrichtung/Position beeinflusst	○	○	○	○	○	○

Seite 14

DG07

14. Wie umfangreich schätzen Sie Ihre Programmierkenntnisse ein? Wählen Sie die für Sie am ehesten zutreffende Option aus.

- ○ Ich habe mich noch nie mit Programmierung beschäftigt
- ○ Installation und Programmierung von Fernsehern oder Handys machen mir keine Probleme
- ○ In meiner Ausbildung hatte ich mit Programmierung in z.B. SPSS, Excel oder höheren Programmiersprachen (z.B. Python, Java, C++) zu tun
- ○ Ich programmiere regelmäßig in SPSS oder Excel
- ○ Ich verwende regelmäßig höhere Programmiersprachen wie Python, Java oder C++

- ○ Keine Antwort

Seite 15

DG08

15. „Digitalisierung in der Medizin“ in der medizinischen universitären Ausbildung. Beurteilen Sie bei den folgenden Aussagen, wie stark Sie diesen zustimmen.

	stimme gar nicht zu	stimme wenig zu	stimme mittelmäßig zu	stimme überwiegend zu	stimme völlig zu	Keine Antwort
Es ist notwendig, technologische Grundlagen der Digitalisierung als Teil des Pflicht-Curriculums zu lehren	○	○	○	○	○	○
Wichtig ist, Wissen über konkrete digitale Anwendungen als Teil des Pflicht-Curriculums zu lehren	○	○	○	○	○	○
Lehrveranstaltungen zur Digitalisierung sollten nur als Wahlfach für Interessierte angeboten werden	○	○	○	○	○	○
Das schulische Allgemeinwissen ist vollkommen ausreichend, es sind keine spezifischen Lehrangebote notwendig	○	○	○	○	○	○

Seite 16

DG09

16. „Digitalisierung in der Medizin“ in der medizinischen universitären Ausbildung. Beurteilen Sie bei den folgenden Aussagen, wie stark Sie diesen zustimmen.

	stimme überhaupt nicht zu	stimme teilweise zu	neutral	stimme größtenteils zu	stimme vollkommen zu	keine Antwort
Ich plane mich mit Digitalisierung in der Medizin zu beschäftigen, auch wenn es nicht Teil des Pflichtcurriculums ist	○	○	○	○	○	○
Ich bin daran interessiert, digitale Produkte in der klinischen Routine einzusetzen	○	○	○	○	○	○
Ich bin daran interessiert, gemeinsam mit Informatikern Anwendungen in der Digitalisierung zu entwickeln	○	○	○	○	○	○

Letzte Seite

Vielen Dank für Ihre Teilnahme!

Wir wünschen Ihnen eine spannende Vortragsreihe und bedanken uns herzlich für Ihre Teilnahme!

Ihre Antworten wurden gespeichert, Sie können das Browser-Fenster nun schließen.
